# Aberdeen Shows the Way: One Day a Week off Duty

**Published:** 1918-10-05

**Authors:** Evelyn Hill

**Affiliations:** Lady Superintendent. The Royal Aberdeen Hospital for Sick Children, Aberdeen


*To the Editor of* The Hospital.

### Aberdeen Shows the Way: One Day a Week Off Duty.

*To the Editor of* The Hospital.

Sir,—As an item of interest for your articles "Round the Hospitals," I am sure you will be pleased to hear that our board of directors has granted such an increase of staff as will enable me to give each sister and nurse a weekly day off duty. At our monthly meeting I brought up for their consideration the long working hours of our nurses and the increased efficiency that would surely result from such a change, and I am happy to say my request was granted with unanimity. And this is, I believe, the first hospital in Scotland to give its nurses this boon.

I have discussed this matter with various other matrons, and everywhere one finds a general disposition to introduce the weekly day off; but lack of accommodation for the necessary increase of staff stands in the way.

The Royal Hospital for Sick Children is a very old and rambling building full of corners and inconveniences, but fortunately for its staff it can still house a few extra nurses, and after the war there is every hope of a new and up-to-date building. The site has been obtained, and a large part of the necessary funds subscribed, and for the remainder it has been suggested that Aberdeen could make no worthier thankoffering for the blessings of peace than to erect a new hospital for its children.

I shall be glad if you can find room for this in your valuable paper.—Yours faithfully, Evelyn Hill, Lady Superintendent.

The Royal Aberdeen Hospital for Sick Children, Aberdeen, September 27, 1918.

* * * We cordially endorse Miss Evelyn Hill's hope for a new hospital for Aberdeen's children after the war.—Ed. The Hospital.

